# Prevalence of Cystic Echinococcosis Genotypes in Iranian Animals: A Systematic Review and Meta-Analysis

**DOI:** 10.1155/2022/8197741

**Published:** 2022-10-19

**Authors:** Sahar Khodashenas, Mehran Akbari, Reza Beiranvand, Mojtaba Didehdar, Mohammad Shabani, Parnia Iravani, Behnam Abedi

**Affiliations:** ^1^Department of Medical Mycology, School of Medicine, Jundishapur University of Medical Sciences, Ahvaz, Iran; ^2^Department of Nursing, Khomein University of Medical Sciences, Khomein, Iran; ^3^PhD of Epidemiology, Khomein University of Medical Sciences, Khomein, Iran; ^4^Department of Medical Parasitology and Mycology, Arak University of Medical Sciences, Arak, Iran; ^5^Department of Medical Laboratory Sciences, Khomein University of Medical Sciences, Khomein, Iran; ^6^Molecular and Medicine Research Center, Khomein University of Medical Sciences, Khomein, Iran

## Abstract

**Background:**

Cystic echinococcosis is considered a public health problem that if left untreated can have dangerous consequences for the person. The disease is caused by *Echinococcus granulosus sensu lato* larvae. The main risk factors for this parasitic infection are habitat, direct contact with dogs, use of raw vegetables, and use of unwashed vegetables. The most important factors affecting the prevalence of HCD are economic, occupational, agricultural, educational, and factors related to public health and cultural habits of the general public in that geographical area.

**Objectives:**

The purpose of this study was to investigate the prevalence of the types of cystic echinococcosis genotypes (E. granulosus sensu stricto (G_1_-G_3_) and E. Canadensis (G_6_ and G_7_)) in livestock in Iran.

**Method:**

This systematic review was conducted, using Medline/PubMed, Scopus, Web of Sciences, and Google Scholar databases, to identify studies of cystic echinococcosis in animals published from 2010 to April 14, 2021. Finally, 28 studies were selected for meta-analysis, which was analyzed using Stata software version 14. The cystic echinococcosis prevalence with 95% confidence intervals of animals was synthesized using the random effect model. Heterogeneity was evaluated and in cases where the *I*^2^ index was higher than 75%, subgroup analysis was performed according to the types of animals.

**Result:**

The highest prevalence of cystic echinococcosis infection was related to G_1_ genotype (*P* = 0.91 (95% CI = 0.84, 0.97)) and the prevalence was related to G_2_ genotype (*P* = 0.07(95% CI = 0.00, 0.18)). The results of the subgroup analysis showed that in the G_1_ genotype the highest prevalence was observed in Goats and Buffaloes with *P* = 1 (95% CI = 0.96, 1) and *P* = 1 (95% CI = 0.97, 1), in the G_3_ and G_6_ genotypes the highest prevalence was observed in camels with *P* = 0.50 (95% CI = 0.31, 0.69), and *P* = 0.45 (95% CI = 0.22, 0.69), respectively.

**Conclusion:**

The cystic echinococcosis genotypes vary from region to region or from country to country and also from host to host, and according to the results, it should always be stopped in areas where the prevalence of such genomes suitable for livestock as well as human food sources to prevent infection of livestock and thus human exposure to cystic echinococcosis.

## 1. Introduction

Cystic echinococcosis (CE) is a common parasitic infection of humans and animals caused by the larva of *9pt?>Echinococcus granulosus sensu lato* and cystic echinococcosis is a widespread zoonotic disease of global concern This disease has been reported in humans from all parts of Iran [1–3], and Cystic echinococcosis is considered an endemic chronic disease that is seen in many countries of the world [4].

Cystic echinococcosis is considered a deadly disease that if left untreated can have dangerous consequences for the person. The disease is caused by Echinococcus granulosus larvae [5, 6].

Echinococcus granulosus is a broad hermaphroditic worm with three growth stages. The structure of a cyst usually consists of three components, which include the pericyst, made of the host's inflammatory tissue, the exocyst, and the endocyst, where the scolex and the prologue membrane are produced [5, 7].

The annual global infection rate is 1.2 million people, the annual mortality rate is about 2.2%, and an estimated 3.6 million disability-adjusted life years (DALYs) are lost annually due to the disease [8].

Echinococcosis/hydatidosis has a global geographical distribution and is observed in all countries. In parts of Eurasia, Africa, Australia, and South America, obscenity is prevalent. E. multilocularis is also distributed in the Northern Hemisphere, including native areas of Central Europe, most of northern and central Eurasia, parts of North America, and North Africa (Tunisia). Epidemiology and control of hydatidosis are often done and since this disease can be known as a disease of livestock, control, and screening of livestock from this infection should be done [9].

Hydatidosis has a global distribution with an annual global incidence rate of 1 to 200 per 100,000. In Iran, hydatidosis is actively transmitted and its annual incidence is estimated at 0.61 per 100,000 [10].

Annual cystic echinococcosis infection causes a lot of economic damage to countries around the world. Infection of livestock with hydatidosis usually leads to a significant reduction in livestock products (meat, milk, and wool) and causes the seizure of infected organs during slaughter. The prevalence of cystic echinococcosis in slaughtered animals in different provinces of Iran is 1.5 to 7. Percentage reported. This cyst can grow in different parts of the animal's body, the most important organs being the liver and lungs. It is worth mentioning that infection with this disease has been mentioned in different parts of Iran, but accurate and comprehensive information on the prevalence of this parasite in livestock and humans is not available at the same time [10].

In a cross-sectional study, a total of 5,381 animals were slaughtered in western Iran, a total of 928 cows, 243 buffaloes, 3,765 sheep, and 445 goats were slaughtered, which were examined macroscopically for cystic echinococcosis. The presence of this parasite was recorded in cows, buffaloes, sheep, and goats with prevalence rates of 38.3%, 11.9%, 74.4%, and 20%, respectively. Prevalence was higher in females than males, but a significant difference (*P* < 0.001) was observed only in sheep and cattle. The majority of convicted cases were observed in sheep lungs (13.4), indicating that sheep are the most important intermediate hosts for *Echinococcus granulosus sensu lato* in this region [11]. Since the general prevalence of this parasite in different types of animals native to Iran is not known, therefore, the aim of this study was to investigate the prevalence of cystic echinococcosis genotypes (E. granulosus sensu stricto (G_1_ (sheep strain), G_2_ (Tasmanian sheep strain), and G_3_ (buffalo strain)), E. Canadensis (G_6_ (camel strain) and G_7_ (pig strains)) [12–14] in Iranian livestock.

## 2. Materials and Methods

### 2.1. Research Question

The purpose of this study was to investigate the prevalence of types of cystic echinococcosis genotypes in livestock in Iran.

### 2.2. Research Strategy

This systematic review was conducted, using Medline/PubMed, Scopus, Web of Sciences, and Google Scholar databases, to identify studies published on hydatid cysts (cystic echinococcosis) in an animal. The keywords used to search the studies were: hydatid cyst, cystic echinococcosis, *Echinococcus granulosus sensu lato,* Animal, Prevalence, Frequency, and Incidence. All relevant keywords were used to search the databases. In order to perform a more comprehensive search, using “and/or”, the above terms were combined.

### 2.3. Inclusion and Exclusion Criteria

The inclusion criteria of the present research consisted of all original articles reporting the prevalence or frequency of cystic echinococcosis (hydatid cysts), either in English or Persian language and published from 2010 to April 14, 2021. Studies were excluded with incomplete information, from other countries, studies that were about human contamination, review articles, opinions, and letters.

### 2.4. Data Extraction and Quality Assessment

**Figure 1:**
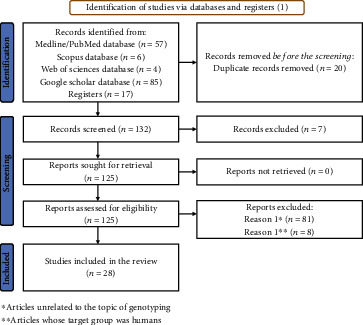
PRISMA flow diagram of the literature search and study selection.

The studies were entered into the EndNote Software for assessment, then, the extraction of data was conducted. The results were reviewed by two authors (conventional double screening), the abstracts were screened, and related studies were selected. All disagreements were resolved through discussion with a third party. Finally, the full texts of the selected studies were reviewed and 28 publications were selected for the meta-analysis (Figure 1). The STROBE (Strengthening the Reporting of Observational Studies in Epidemiology) was used to determine the quality of the studies [13]: (i) inclusion and exclusion criteria, (ii) methods of selection of participants, (iii) definition of the outcome, (iv) definition of exposure, and (v) calculation of the sample size. Studies with five-star items were considered high-quality studies, and those with four star-items or less were considered low-quality studies.

Finally, a checklist was prepared by the research team to extract the variables of sample size, type of animal, study location, infection prevalence, and type of genotype for meta-analysis and also subgroup analysis.

### 2.5. Statistical Analyses

The current meta-analysis was executed using Stata software version 14 (StataCorp. 2015, Stata Statistical Software: Release 14, College Station, TX). The cystic echinococcosis with the prevalence of animals was synthesized using the random effect model. Heterogeneity was evaluated with the Q test and the *I*^2^ index. Studies with an *I*^2^ index of <25%, 25–75%, and>75% fell into the category of low, moderate, and high heterogeneity, respectively. In cases where the *I*^2^ index was higher than 75%, subgroup analysis was performed according to the types of animals studied. Forest plots were used to visualize the prevalence in each study and the incorporated estimated with 95% confidence intervals (95% CI), both in the main analysis and the subgroup analysis.

## 3. Result

A total of 152 records were identified in databases, during the initial search. We identified 57 papers on Medline/PubMed, 6 papers from Scopus, 4 Papers from Web of Sciences, and 85 papers from Google Scholar. After removing duplicates and applying our exclusion criteria, a title and abstract analysis were performed for 125 papers. Only 28 papers included the relative frequency percentage of cystic echinococcosis in animals. The 28 papers underwent the quality assessment and were included in our meta-analysis (Figure 1).

**Figure 2:**
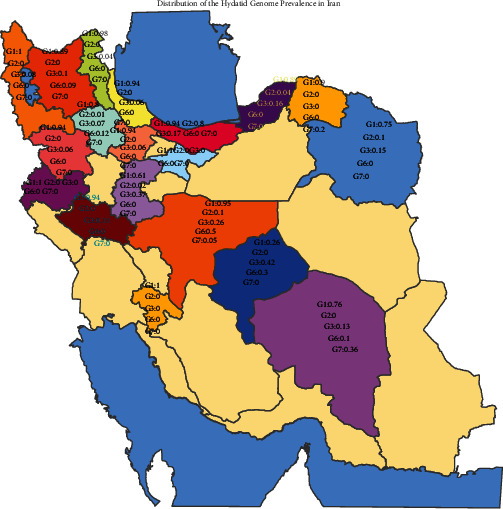
Distribution of different types of cystic echinococcosis genotypes in Iran.

Based on the drawn GIS map, it was found that the frequency of different types of cystic echinococcosis genotypes is higher in the western and northwestern regions of Iran, which is a mountainous region and livestock farming is more prevalent (Figure 2).

**Figure 3:**
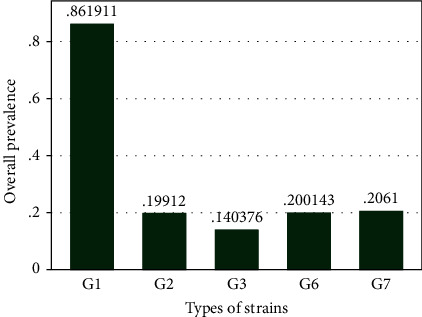
Overall prevalence of different types of cystic echinococcosis genotypes in Iran (Regardless of their weight in meta-analysis).

Based on the drawn bar chart, regardless of the weight of the studies in the meta-analysis, G_1_ genotype had the highest prevalence and G_3_ genotype had the lowest prevalence (Figure 3).

### 3.1. Baseline Characteristics of Studies

Table 1 shows the final information of the studies included in the meta-analysis. To have a well-defined outcome and the ability to perform a meta-analysis, analysis was performed in the genotype groups G_1_, G_2_, G_3_, G_6_, and G_7_. Also, the type of animal under study (Camel, Sheep, Cattle, Goat, Buffalo, Dog, Red Fox, Jackal, and Donkey) and the sample size of each animal in each study were extracted.

The data obtained from all the studies that entered the analysis phase showed that 2579 animals were examined, of which the most studied animals in different studies were sheep (37.6%), and the lowest sample size belonged to Jackal and Donkey (0.05). %) (Table 2).

### 3.2. Main Analysis

**Figure 4:**
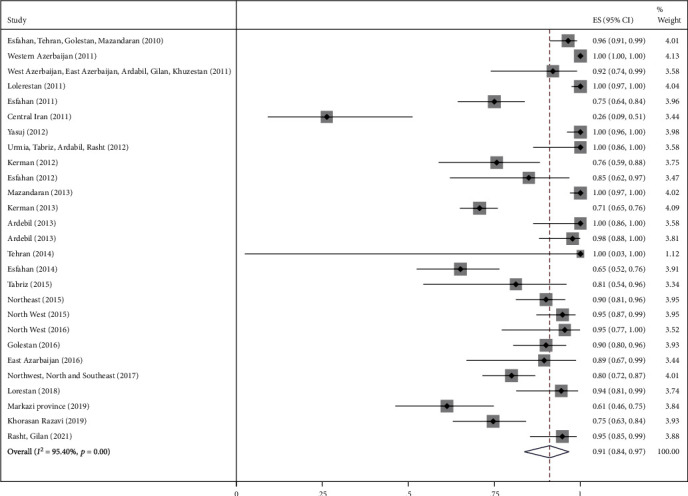
Forest plot of 27 studies on cystic echinococcosis G_1_ genotype prevalence in animals.

**Figure 5:**
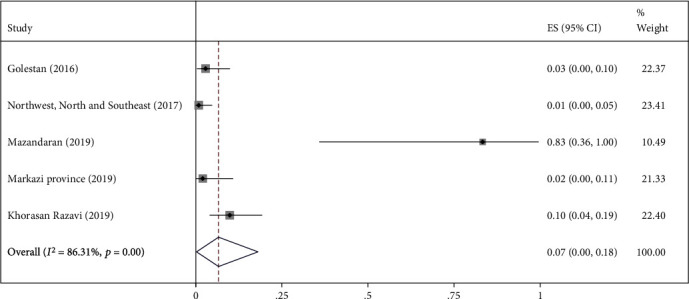
Forest plot of 5 studies on cystic echinococcosis G_2_ genotype prevalence in animals.

**Figure 6:**
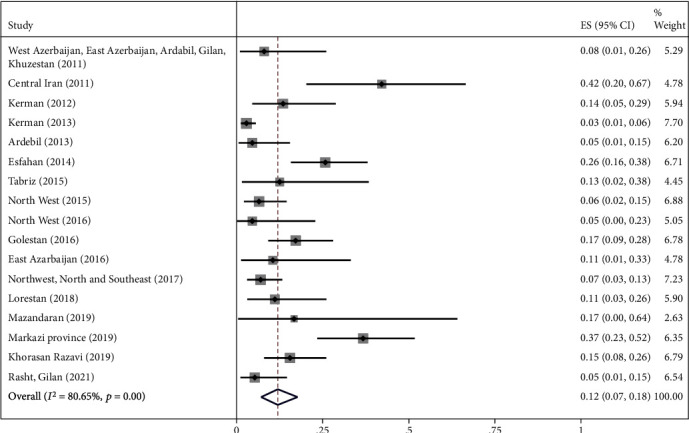
Forest plot of 17 studies on cystic echinococcosis G_3_ genotype prevalence in animals.

**Figure 7:**
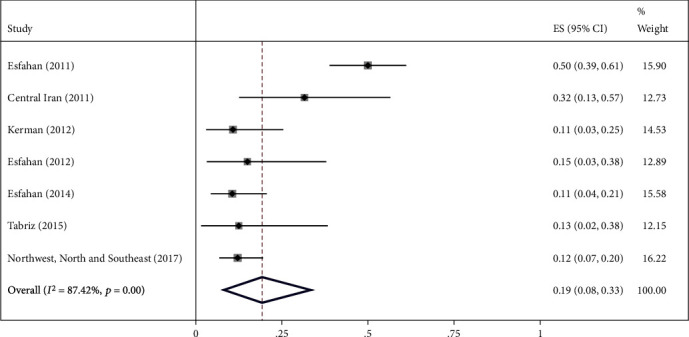
Forest plot of 7 studies on cystic echinococcosis G_6_ genotype prevalence in animals.

**Figure 8:**
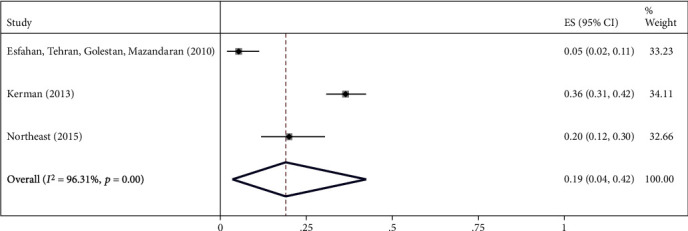
Forest plot of 3 studies on cystic echinococcosis G_7_ genotype prevalence in animals.

The highest relative frequency percentage of infection with cystic echinococcosis in animals was related to G_1_ genotype with *P* = 0.91 (95% CI = 0.84, 0.97) and the lowest relative frequency percentage was related to G_2_ genotype with *P* = 0.07 (95% CI = 0.00, 0.18). Pooled estimates of infection relative frequency percentage were also statistically significant in G_3_, G_6_, and G_7_ genotypes (Tables 3 and Figures 4–8).

### 3.3. Subgroup Analysis

**Figure 9:**
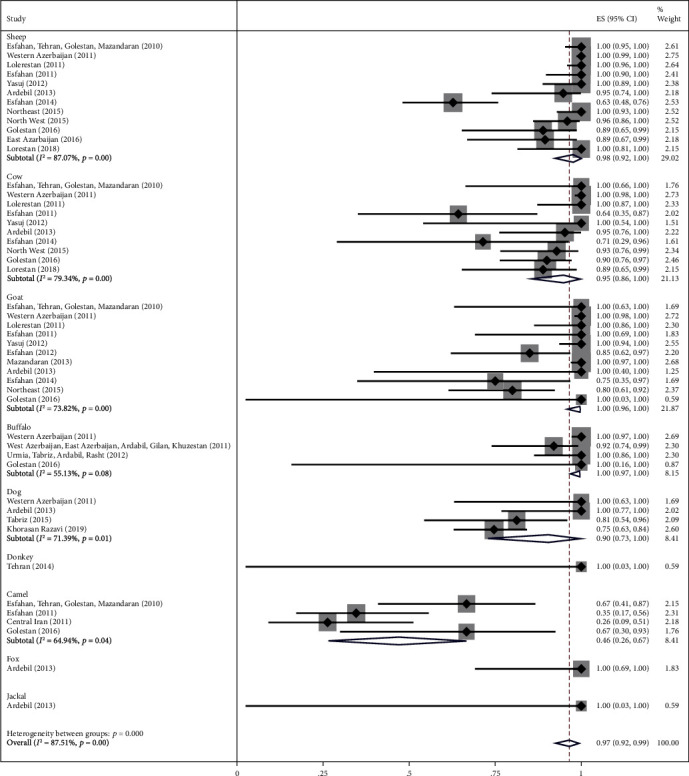
Forest plot of subgroup analysis of studies on cystic echinococcosis G_1_ genotype prevalence by animal types.

**Figure 10:**
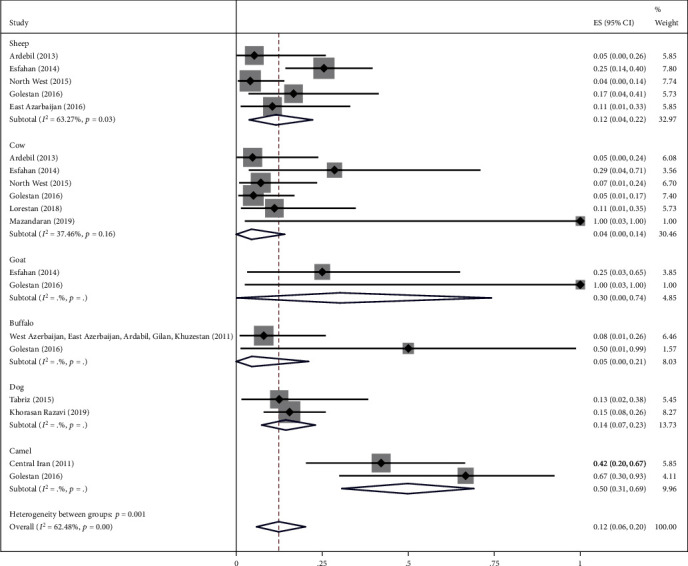
Forest plot of subgroup analysis of studies on cystic echinococcosis G_3_ prevalence by animal types.

**Figure 11:**
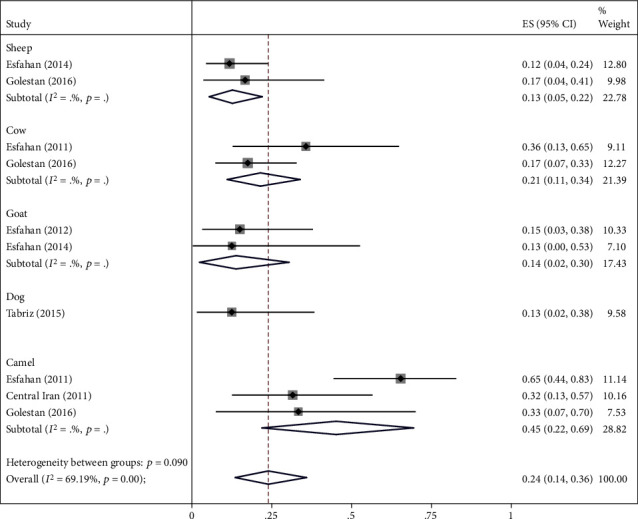
Forest plot of subgroup analysis of studies on cystic echinococcosis G_6_ genotype prevalence by animal types.

To reduce heterogeneity in the pooled estimation of the relative frequency percentage of cystic echinococcosis, in G_1_, G_3_, and G_6_ genotypes, subgroup analysis was performed based on the type of animal under study. The results showed that in G_1_ genotype the highest relative frequency percentage was in Goat and Buffalo with *P* = 1 (95% CI = 0.96, 1) and *P* = 1 (95% CI = 0.97, 1), respectively, in G_3_ and G_6_ genotype. In camels, it was obtained with *P* = 0.50 (95% CI = 0.31, 0.69) and *P* = 0.45 (95% CI = 0.22, 0.69), respectively. Subgroup analysis was not possible in G_2_ and G_7_ genotypes due to the low sample size (Table 4 and Figures 9–11).

## 4. Discussion

The distribution of cystic echinococcosis livestock genotypes in Iran is different from each region to another region, as well as from host to host. Depending on the type of climate and vegetation in Iran, different domestic animals are kept as livestock by the people, since livestock and animal husbandry are traditionally practiced in most parts of Iran. The traditional method of care exposes livestock to parasitic infections, including cystic echinococcosis; therefore, despite initial studies, the prevalence of genotypes of this parasite, especially by animal type, remained unclear. This systematic review and meta-analysis study was performed to investigate the prevalence of hydatid cyst in livestock in Iran. Preliminary study data published from 2010 to April 14, 2021, were collected and analyzed. Finally, 28 studies had the eligibility criteria of the present study and their information was extracted based on the type of animal under study and their cystic echinococcosis genotype. The most studied animal species were sheep and then goats and the most abundant genotype found was G_1_.

The results of the meta-analysis of our study showed that the most common genotypes of cystic echinococcosis in animals in Iran, were *Echinococcus granulosus sensu lato* (sheep genotype or G_1_) and E. Canadensis group (camel genotype or G_6_), respectively. Canadensis group (Pig genotype or G_7_) were E. The results of the study of Tiemin Zhang et al. showed that the most abundant genotype of cystic echinococcosis in animals of China is G_1_ and then G_6_ [42], also genotype G_1_ is the most abundant type of cystic echinococcosis in different hosts in Ethiopia [43], Tunisia [44], Palestine [45], India [46], China [47], Mongolia [48], and Turkey [49] which is consistent with the results of the present study, in the study of Kheirandish et al., in Lorestan province [40], and the study of Asma A. Latif et al., in Pakistan [50] showed that after G_1_ was identified as the most abundant cystic echinococcosis genotype of G_3_. However, in the study of Said Amer et al., in Egypt [51], the most abundant genotype extracted was G_6_. Also, the study of Shahnazi et al., in Isfahan province [16] and the study of Sharbatkhori et al., in central Iran [34] in isolated samples of camels was the most abundant G_3_ genotype, which can be due to different studies in different geographical areas. Cystic echinococcosis may be present during sampling and testing, as well as differences in tissue.

The results of subgroup analysis showed that the highest infection in the G_1_ genotype was related to Goat samples (*P* = 1, 95% CI = 0.96, 1) and Buffalo (*P* = 1, 95% CI = 0.97, 1); the results of the Pezeshki et al., study in Ardabil province in northern Iran [32], showed that more than 90% of the infections in Goat and Sheep were of G_1_ genotype, which is almost similar to our study and in the study of Hajialilo et al., in the southeast of Iran [33], the results showed that 100% of the samples isolated from Goat were infected with the G_1_ genotype.

The results of our study subgroup analysis showed that the highest infection with G_3_ genotype was seen in cysts isolated from Camel with *P* = 0.50 (95% CI = 0.31, 0.69). In the study of Abd El Baki et al., in Golestan province in northern Iran [52], the frequency of G_3_ genotype in cysts isolated from Camel was 66.7%, and in the study of Sharbatkhori et al., in central Iran [34] the relative frequency percentage of G_3_ genotype in cysts Camel was 42.1%. While the results of a study by Abd El Baki et al., [52] showed that the most common genotype in Camel was the G_1_ genotype with a 90% relative frequency percentage, the reason for this discrepancy could be the existence of the sheep breeding industry in Egypt. The use of Camels as a means of public transportation in desert areas and close contact between the two, which eventually led to the predominance of the genotype.

In the study of Sharbatkhori et al., in central Iran [34] in cysts isolated from Camel, 31.6% infection was seen in the G_6_ genotype, and in the study of Shahnazi et al., in Isfahan province [16] 65.36% of the samples isolated from Camel, genotype G_6_, while the results of our subgroup analysis showed that the highest percentage of infection with G_6_ genotype was seen in cysts isolated from Camel with *P* = 0.45 (95% CI = 0.22, 0.69); however, in the study of Said Amer et al. [51], the highest percentage of G_6_ genotype infection was observed in camels with 0.92%, which was much higher than the percentage of infection in our study. The reason for the mismatch could be camel meat as a rich source of Protein in Egypt, which has led to the high relative frequency percentage of Camel genotype in recent years in this region.

## 5. Conclusion

The results of our article showed that the distribution and prevalence of Echinococcus genotypes varies from region to region, or from country to country, and also from host to host, and in Iran due to climate and vegetation, its prevalence in different species. The most studied animal species were sheep and then goats and the most abundant genotype was G_1_. The results showed that the most common genotypes of cystic echinococcosis in Iranian animals were sheep, camel, and pig genotypes, respectively, which according to the results, should it seem that in the areas where the CA method is high, the necessary steps should be taken regarding the identification and timely treatment of livestock in order to prevent the spread of this disease in animals and ultimately humans.

## Figures and Tables

**Table 1:** Baseline characteristics of the 28 studies included in the meta-analysis of cystic echinococcosis relative frequency percentage in animals.

	Geographical region	Type of animals	Sample size	Cystic echinococcosis genotype [1]					References
				G_1_ (sheep strain)	G_2_ (Tasmanian sheep strain)	G_3_ (buffalo strain)	G_6_ (camel strain)	G_7_ (pig strain)	
1	Lolerestan (2011)	Sheep	88	100%					[15]
		Cattle	27	100%					
		Goat	25	100%					
2	Esfahan (2011)	Camel	26	34.61%			65.39%		[16]
		Cattle	14	64.28%			35.71%		
		Sheep	34	100%					
		Goat	10	100%					
3	Western Azerbaijan (2011)	Sheep	270	100%					[17]
		Goat	185	100%					
		Cattle	197	100%					
		Buffalo	129	100%					
		Dog	8	100%					
4	Urmia, Tabriz, Ardabil, Rasht, Ahvaz (2012)	Buffalo	25	100%					[18]
5	Esfahan (2012)	Goat	20	85%			15%		[19]
6	Yasuj (2012)	Sheep	31	100%					[20]
		Goat	56	100%					
		Cattle	6	100%					
7	Ardebil (2013)	Dog	14	100%					[21]
		Red fox	10	100%					
		Jackal	1	100%					
8	Mazandaran (2013)	Goat	120	100%					[22]
9	Tehran (2014)	Donkey	1	100%					[23]
10	East Azarbaijan (2016)	Sheep	19	89.47%		10.52%			[24]
11	Tabriz (2015)	Dog	16	81.25		9.37%	9.37%		[25]
12	Northwest (2015)	Sheep	49	96%		4.00%			[26]
		Cattle	28	92.85%		7.15%			
13	Mazandaran (2019)	Sheep	5		100%				[27]
		Cattle	1			100%			
14	Golestan (2016)	Camel	9	66.70%	66.70%	66.70%	33.30%	33.30%	[28]
		Sheep	18	89.21%		3.07%	16.92%		
		Cattle	40	89.21%		3.07%	16.92%		
		Buffalo	2	100%		50%			
		Goat	1	100%		100%			
15	Northwest, North, and Southeast (2017)	—	115	80%	0.86%	9.6%	12.17%		[29]
16	Northeast (2015)	Sheep	50	100%					[30]
		Goat	30	80%				20%	
17	Esfahan (2014)	Sheep	51	63.00%		25.00%	12%		[31]
		Goat	8	77%		21%	2%		
		Cattle	7	72%		28%			
18	Ardebil (2013)	Sheep	19	95%		5%			[32]
		Goat	4	100%					
		Cattle	21	99.95%		4.76%			
19	Kerman (2012)	—	37	75.67%		13.51%	10.81%		[33]
20	Central of Iran (2011)	Camel	19	26.30%		42.10%	31.60%		[34]
21	West Azerbaijan, East Azerbaijan, Ardabil, Gilan, Khuzestan (2011)	Buffalo	25	92%		8%			[35]
22	Northwest (2016)	—	22	94.50%		5.70%			[36]
23	Kerman (2013)	—	280	70.76		3%		36.5%	[37]
24	Rasht, Gilan (2021)	—	57	94.20%		5.80%			[38]
25	Esfahan (2010)	Sheep	37	100%					[39]
		Cattle	4	100%					
		Camel	18	66.66%				33.33%	
		Goat	8	100%					
	Tehran (2010)	Sheep	27	100%					
	Golestan (2010)	Sheep	10	100%					
		Cattle	5	100%					
	Mazandaran (2010)	Sheep	3	100%					
26	Markazi province (2019)	—	49	61%	2%	37%			[6]
27	Lorestan (2018)	Sheep	18	100%					[40]
		Cattle	18	88.90%		11.10%			
28	Khorasan Razavi (2019)	Dog	71	75%	10%	15%			[41]

**Table 2:** Type of animals percent in 28 studies included in the meta-analysis.

Type of animals	Number	Valid percent (%)
Camel	72	3.72
Sheep	729	37.62
Cattle	369	19.04
Goat	466	24.05
Buffalo	181	9.34
Dog	109	5.62
Red fox	10	0.51
Jackal	1	0.05
Donkey	1	0.05
Not defined	638	—
Total	2576	—

**Table 3:** The pooled estimate of cystic echinococcosis genotypes prevalence in animals of Iran.

Type of genotype	Number of studies in which each genotype was evaluated	Number of participants	Random pooled ES (95% CI)	*P* value for test (ES = 0)	*I* ^2^ (%)	The *P* value for the heterogeneity test	Estimate of between-study variance (Tau^2^)
G_1_	27	2317	0.91 (0.84,0.97)	<0.001	95.40%	<0.001	0.25
G_2_	5	17	0.07 (0.00, 0.18)	0.02	86.31%	<0.001	0.11
G_3_	17	122	0.12 (0.07, 0.18)	<0.001	80.65%	<0.001	0.07
G_6_	7	74	0.19 (0.08, 0.33)	<0.001	87.42	<0.001	0.15
G_7_	3	156	0.19 (0.04, 0.42)	<0.001	96.31%	<0.001	0.20

**Table 4:** Subgroup analysis of cystic echinococcosis genotypes prevalence by animal types.

Type of genotype		Number of studies in which each genotype was evaluated	Random pooled ES (95% CI)	*P* value for test (ES = 0)	*I* ^2^ (%)	The *P* value for the heterogeneity test
Type of animals						
G1	Sheep	12	0.98 (0.92, 1)	<0.001	87.07	<0.001
	Cattle	10	0.95 (0.86, 1)	<0.001	79.34	<0.001
	Goat	11	1 (0.96, 1)	<0.001	73.82	<0.001
	Camel	4	0.46 (0.26, 0.67)	<0.001	64.94	0.04
	Buffalo	4	1 (0.97, 1)	<0.001	55.13	0.08
	Dog	4	0.90 (0.73, 1)	<0.001	71.39	0.01
	Red fox	1	1 (0.69, 1)	<0.001	—	—
	Jackal	1	1 (0.03, 1)	0.05	—	—
	Donkey	1	1 (0.03, 1)	0.05	—	—
	Overall	48	0.97 (0.92, 0.99)	<0.001	87.51	<0.001
G3	Sheep	5	0.12 (0.04, 0.22)	<0.001	63.27	0.03
	Cattle	6	0.04 (0.00, 0.14)	0.06	37.46	0.16
	Goat	2	0.30 (0.00, 0.74)	0.04	—	
	Camel	2	0.50 (0.31, 0.69)	<0.001	—	
	Buffalo	2	0.05 (0.00, 0.21)	0.22	—	
	Dog	2	0.14 (0.07, 0.23)	<0.001	—	
	Overall	19	0.12 (0.06, 0.20)	<0.001	62.48	<0.001
G6	Sheep	2	0.13 (0.05, 0.22)	<0.001	—	—
	Cattle	2	0.21 (0.11, 0.34)	<0.001	—	—
	Goat	2	0.14 (0.02, 0.30)	<0.001	—	—
	Camel	3	0.45 (0.22, 0.69)	<0.001	—	—
	Dog	1	0.13 (0.02, 0.38)	0.03	—	—
	Overall	10	0.24 (0.14, 0.36)	<0.001	69.19	<0.001
